# Survival from endometrial cancer in England and Wales up to 2001

**DOI:** 10.1038/sj.bjc.6604592

**Published:** 2008-09-23

**Authors:** H C Kitchener

**Affiliations:** 1 School of Cancer and Imaging Sciences, University of Manchester, Research Floor, St Mary's Hospital, Whitworth Park, Manchester M13 0JH, UK

## Clinical presentation

Endometrial cancer is rare in premenopausal women, usually presenting with postmenopausal bleeding. This alarming symptom prompts most affected women to present to their general practitioner, which results in urgent referral to a gynaecological unit. In premenopausal women, the usual menstrual pattern may be disturbed, but irregular bleeding is common in the fifth decade and referral may not be so prompt. If bleeding as an early sign is disregarded, the tumour will progress; enlargement and spread to the pelvic sidewall can result in pain.

## Diagnosis and treatment

The mainstay of diagnosis is a biopsy, obtained usually as an outpatient under direct hysteroscopic vision or using a suction instrument, such as a Pipelle, inserted through the cervical os. As only around 8–10% of women who experience postmenopausal bleeding actually have cancer, it is currently recommended that women be screened initially with ultrasound of the uterus, which clearly demonstrates the thickness of the endometrium. It has been determined from a number of large studies that endometrial tissue measuring less than 5 mm thickness, has a negative predictive value of 99%, thus avoiding the need for routine biopsy. The current standard is to establish a ‘one-step’ rapid access clinic, which combines clinical evaluation, ultrasound, and if necessary, an endometrial biopsy with or without hysteroscopic exploration of the endometrial cavity.

Complete assessment of an endometrial cancer requires the histotype to be determined; although most are endometrioid, rarer types such as clear-cell, papillary serous and carcinosarcoma, each of which account for around 2–4% of the total, have a significantly worse prognosis because of a more frequent rate of metastases at presentation and a higher recurrence rate.

Treatment has not changed a great deal over the past 30 years, although a number of clinical trials are beginning to inform management in a more rational way. For early-stage disease, which accounts for 80–90% of cases, management has relied on hysterectomy and bilateral salpingo-oophorectomy followed by pelvic radiotherapy if the tumour histology indicated a high-grade tumour and/or deep myometrial invasion. Although pelvic radiotherapy reduces the risk of local relapse, there is no evidence that it affects overall survival and increasingly, systematic therapy (chemotherapy) is being evaluated for high-risk or metastatic disease.

## Interpretation of survival patterns

The incidence of endometrial cancer has risen in the United Kingdom as a result of increasing rates of obesity, increased life expectancy and the use of tamoxifen as an adjuvant therapy for breast cancer. Disease occurring as a result of obesity and tamoxifen is generally detected at an early stage and often is well differentiated leading to high disease-specific survival. Enhanced heath awareness and rapid systems of referral and diagnosis may also be implicated in the slightly higher proportion of early-stage disease. In addition, there may have been some disease shift as a result of the increased practice of lymphadenectomy to stage the disease. This means that disease classified as stage I is more likely to be truly confined to the uterine corpus than disease only defined by hysterectomy and bilateral salpingo-oophorectomy, in which a small proportion will have occult node involvement, and should be classified as stage 3.

It may be that the small increase in survival over the past 10–15 years is due to a combination of these factors, and to optimal treatment in a higher proportion of cases, as more women are treated by specialist teams.

## Deprivation gap

The small but real difference in survival according to the deprivation index may be best explained by comorbidity. Women with endometrial cancer have relatively high rates of obesity, hypertension, coronary artery disease, diabetes and chronic respiratory disease. This complex of comorbidity is likely to be more common among women living in deprived areas. A significant proportion of deaths within 5 years of endometrial cancer being diagnosed is due to other causes but may include endometrial cancer on the death certificate. It is possible that deprivation is associated with delayed diagnosis but this is a less likely explanation. Gross obesity, which is increasingly common may not only be the cause of the disease but can preclude curative surgery and in a small number of cases results in suboptimal treatment by brachytherapy alone.

## Closing the deprivation gap

There is no reason why deprivation should now have a detrimental effect on the quality of cancer care for endometrial cancer. The means of closing the deprivation gap may lie in a general improvement in health indices so that women who are cured of endometrial cancer have a high prospect of prolonged survivorship. Reduction in obesity and smoking will be major factors in achieving this.

## Overall comment

Although survival in endometrial cancer is higher than many other cancers including the other gynaecological cancers there is a need to save more lives. This may be achievable by changes in lifestyle (especially obesity) but in the absence of a secondary prevention strategy there needs to be improvement in the treatment of advanced disease, which is currently difficult to cure. We will see more trials of chemotherapy both cytotoxic and biological agents, for example, antiangiogenic agents, over the next 10 years, which will demonstrate whether greater cure rates are possible.

